# Strengthening Global Legislative Actions to Protect Children from the Harmful Impacts of Unhealthy Food and Non-alcoholic Beverage Marketing

**DOI:** 10.1007/s13679-023-00492-6

**Published:** 2023-02-13

**Authors:** Fiona Sing, Kathryn Backholer

**Affiliations:** 1 Department of Medicine and Health Sciences, School of Population Health, University of Auckland, 85 Park Road, Grafton, Auckland, New Zealand; 2 Global Centre for Preventive Health and Nutrition, Institute for Health Transformation, Deakin University, Geelong, Australia

**Keywords:** Marketing, Children, Legislation, Food policy, Food law, Regulatory design

## Abstract

**Purpose of Review:**

Drawing on current global food marketing policies and the academic literature, we illustrate why and how existing legislative approaches can be strengthened to better achieve the goal of protecting children from the harmful impacts of unhealthy food and non-alcoholic beverage marketing.

**Recent Findings:**

Around the world, governments are starting to implement legislative actions to protect children from exposure to unhealthy food and non-alcoholic beverage marketing. However, the scope of these approaches is limited and unlikely to meet their objective of safeguarding children from harmful marketing practices. The key opportunities for strengthening legislative design include increasing the age threshold of a child to 18 years; the broadening of focus from ‘child-directed’ marketing to all marketing that children are exposed to; designing tailored regulations for multiple settings, media and techniques; strengthening underlying food classification systems; balancing a comprehensive approach with stepwise regulatory implementation; and strengthening monitoring and enforcement systems.

**Summary:**

Our review and recommendations can aid policymakers when designing legislative approaches to protect children from the harmful impacts of unhealthy food and non-alcoholic beverage marketing.

## Introduction

Globally, the prevalence of overweight and obesity is at unacceptable and unsustainable levels. In 2020, 39 million children under 5 years of age experienced overweight or obesity, and in 2016, over 340 million children and adolescents aged 5–19 were living with overweight or obesity [1]. Overweight and obesity are driven, predominantly, by a shift towards highly processed, unhealthy, diets that are high in saturated fats, trans-fatty acids, free sugars or salt [2]. Despite the known health risks associated with these foods and beverages, children’s exposure to unhealthy food marketing is ubiquitous [3–5]. Across the life course, this creates social norms and increases preference and consumption of unhealthy foods, leading to excess weight gain and an increased risk of overweight and obesity, cognitive impairments, reduced quality of life and non-communicable disease [6–9]. UN agencies such as the World Health Organisation (WHO) and the UN Commission on Human Rights have called on Member States to restrict children’s exposure to unhealthy food and beverage marketing [6, 10–13, 14•, 15–17]. While some governments have introduced a regulatory approach to address this issue, very few countries have laws in place that adequately protect *all* children from *all* unhealthy food and beverage marketing [13, 14•, 18••, 19].

The WHO states that reducing the exposure to, and power of, unhealthy food and beverage marketing to children should be the ultimate goal of any regulatory response [10, 11]. Further to this, Article 24 of the United Nations Convention on the Rights of the Child (UNCRC) has been interpreted as requiring government action to restrict the exposure of children to unhealthy food and beverage marketing [13, 16, 17, 20–23]. Therefore, protecting children from exposure to unhealthy food marketing is a legitimate public health objective; however, it can be a technically difficult area to regulate. Robust policy design is crucial to ensure the marketing restrictions meet this overall policy objective. A law that can be shown to effectively meet its stated objectives and that can be enforced will place governments in a stronger position. Foreseeing and minimising any loopholes or unintended outcomes are an important design challenge. Not only is this important for effective regulatory implementation, but also to ensure governments mitigate any risk of challenge to the regulatory approach by actors who are opposed to restrictions on food marketing.

In this paper, we drew on existing global food marketing policies and the academic literature to illustrate why and how global legislative approaches can be strengthened to better achieve the goal of protecting children from the harmful impacts of unhealthy food and beverage marketing. We examined three key technical areas of a legislative approach to food marketing controls, which were identified as the most pertinent technical issues facing governments in the literature [13, 14•, 18••, 24–30]. These include (i) how to capture the full extent of children’s exposure to unhealthy food and beverage marketing in a comprehensive law (including scope related to the age of children; the marketing types, techniques and channels; brand marketing; tailoring the legal design to different media, settings and marketing techniques; and food or nutrient classification systems); (ii) how to balance a comprehensive legislative approach with practical implementation of a multi-faceted law; and (iii) strengthening monitoring processes and increasing enforcement provisions to implement laws more robustly. In this paper, we outline the current state of regulatory design for each of the areas covered and provide an explanation of why and how the technical approach can be strengthened. This paper can aid policymakers in designing effective and comprehensive laws that adequately protect children from the harmful impacts of unhealthy food and beverage marketing.

## Capturing the Full Extent of Children’s Exposure to Unhealthy Food and Beverage Marketing

### Age of Children

Globally, while some countries, including Ireland, South Korea and Turkey, define children in their food marketing legislation as persons aged up to 18 years, most food marketing policies only protect younger children [13, 14•, 18••, 31].

One of the reasons why older children are not commonly within the scope of international food marketing laws is because they are considered to have greater cognitive capacity than younger children and are viewed as less vulnerable to economic exploitation. However, evidence shows that food marketing also negatively influences adolescents. Adolescents are more likely to be influenced by their peers and are reward driven, impulsive and vulnerable to the promotion of products that provide immediate gratification [32, 33]. They have more purchasing power than younger children with access to their own money [34], and consumption levels of unhealthy foods and beverages are particularly high and concerning from a public health perspective [5, 6].

Governments have also faced challenges when trying to introduce laws to protect children up to age 18 [14•, 24, 27]. For example, during the policy development of a bill to restrict food and beverage advertising to children, the Canadian government reduced the age limit from 17 years old to 13 years old [27, 35]. Reasons for this reduced age threshold may relate to the argument (by those that oppose the legislation) that it would be too hard to discern food marketing that is ‘directed to’ a 17-year-old rather than an 18-year-old [14•, 24–26, 36, 37] or that marketing ‘directed’ or ‘targeted’ at adolescents would be too similar to marketing intended for adults [14•, 24, 37].

Under Article 24 of the UNCRC children are defined as ‘every human being below the age of 18 years unless, under the law applicable to the child, majority is attained earlier’ [38]. This is relevant as the UN Human Rights Council have interpreted a child’s right to health, enshrined in Article 24, as including the protection of children from unhealthy food and beverage marketing [15, 17, 22, 23, 38, 39]. All but two UN Member States have ratified the UNCRC and therefore have the legal obligation to fulfil the right of the child to enjoy the highest attainable standard of health and therefore the obligation to restrict unhealthy food and beverage marketing to children aged 18 years and under [13, 21, 40, 41]. Therefore, given the evidence base that exists to show that adolescents are vulnerable to marketing practices and the UNCRC legally requiring protection of children up to 18, governments should ensure their legal response defines children as ‘up to 18 years of age’.

### Marketing Types, Techniques and Channels in Scope

Most countries focus on restricting food marketing through broadcast channels, particularly child-specific broadcasting (mainly television) [18••, 31] as it is easier to design and enforce compared to other settings and media and is viewed as more politically acceptable [14•]. To protect *all* children from *all* unhealthy food and beverage marketing, it is essential that the legislative scope includes unhealthy food marketing across all media and settings and all marketing techniques that children are exposed to [11, 14•, 42••]. This includes school settings; retail environments (product placements and price promotions in food stores); outdoor advertising (e.g. billboards); marketing in schools (including advertising, promotion and sponsorship); marketing in other children’s settings such as recreational facilities and event venues (including advertising, promotion and sponsorship); marketing online (including social media); advertising in broadcast media (television, cinema and radio); child-directed marketing on food packaging; sponsorship of sports and other major public events; and advertising on public transport (bus shelters, trains) and print media. [11, 14•, 42••].

Several countries have introduced comprehensive food marketing restrictions, across all media and settings, but are limited by a focus on ‘child-directed’ marketing content and exemptions for brand advertising [13, 14•]. In Quebec, Canada, under the Consumer Protection Act, all commercials are prohibited where children (< 13 years) comprise more than 15% of the audience, and the product featured is exclusively designed for, or appeals to, children [43]. In Ireland, the Children’s Commercial Communication Code only applies where commercial communications target children under 18 and are broadcast during programmes where children comprise more than 50% of the total audience [44].

As part of the broadcast aspect of the Food Labelling and Advertising Law, the Chilean Government originally designed their restrictions as any unhealthy food advertising on television programmes considered to be (i) ‘child-targeted’, (ii) where > 20% of the audience consists of children aged < 14 years, and (iii) where advertising appeals to children by including characters, toys or other strategies considered to be ‘directed to children’ [18••, 45]. Research has shown that while a ‘child-targeted’ approach did reduce the exposure of advertising by 35% for pre-schoolers and 52% for adolescents, it did not eliminate television advertising for products subjected to the law [46]. The authors of this study suggest that the reasons for the remaining exposure of unhealthy food and beverage marketing on television is likely a consequence of food marketing outside of child-targeted programming and with the difficulties in interpreting ‘child-directed’ marketing and thus ensuring compliance with the law [46]. In June 2018, the Chilean Government adapted the design of the legal response to replace the ‘child-targeted’ definition with a time-based restriction that banned all unhealthy food advertising on television channels between 6am and 10 pm. While there has been no evaluation of this later policy amendment, it is expected that it will eliminate the vast majority of children’s exposure to unhealthy food advertising on television [46]. Similarly, the UK has recognised the limitations with its original legislative approach to broadcast legislation, which stated ‘no medium should be used to advertise HFSS products, if more than 25% of its audience is under 16 years of age’ and passed legislation that introduced a 6am until 9 pm watershed of unhealthy food and beverage marketing, although implementation has been stalled indefinitely [47, 48].

This approach of restricting unhealthy food marketing that is ‘targeted at’, ‘directed at’, ‘appealing to’, ‘child-directed’, specific to ‘children’s programming’ or specific to ‘children’s media’ is limited for several reasons [13, 14•, 34]. With this approach, governments have to discern exactly what is subject to the legal response. Children’s media or programming (particularly television) is often defined using audience thresholds, using the proportion of children in the audience. This is problematic as many children consume the same media and attend the same settings as adults, so a small proportion may represent a very large number of children [34]. The frequency of these mixed-audience environments increases as children grow older; therefore, the use of audience thresholds becomes increasingly difficult. Similarly, marketing techniques that are defined as being targeted to children are commonly determined by a ruling of whether the marketing is ‘child-appealing’; however, understanding what is ‘child-appealing’ is nuanced and subjective and therefore incredibly difficult to implement and enforce [27, 49]. Therefore, ‘child-directed’ marketing definitions are not sufficient as children are exposed to a wide range of media sources and content that are not specifically ‘targeted’ or ‘directed’ at them [34].

#### Brand Marketing

Brand marketing is a key marketing tactic where a food company advertises their master brand or logo, which emphasises a corporate brand instead of a specific product. However, including brand marketing within the scope of a legal response can be difficult, and globally, there are no legal responses of unhealthy food and beverage marketing that include brand marketing within their scope [31]. This is, in part, because the legal design is underpinned by a nutrient or food classification system, which has been designed to classify foods and beverages as ‘permitted’ or ‘not permitted’ to be marketed, not brands. Therefore, marketing that does not feature a food or beverage product is exempt.

Exemptions of brand marketing are a public health issue as evidence shows that advertising of brands that are primarily associated with unhealthy foods and non-alcoholic beverages (e.g. quick service restaurants or confectionary) increases reward pathways in the brain and selection and consumption of unhealthy products [50, 51]. Similarly, unhealthy food sponsorship (from the local to elite level), which is often reciprocated through the advertisement of sponsors' brands, has been shown to increase brand awareness and preference for branded products [52, 53]. Legal responses that do not incorporate brand marketing may also result in the unintended consequence of shifts in marketing spend from restricted forms of food marketing to unrestricted marketing of brands and logos.

This can be addressed by creating a brand classification system for assessing which brands will be subject to a restriction. For example, a brand’s top five selling items by market share could be identified, and if three or more of these products are not permitted to be marketed according to the legislated food classification system, then any associated brand advertising could also be prohibited.

Only with a broad definition of marketing will every child be given the same level of protection, regardless of how they access their media content. Further, a comprehensive legislative response that covers all types of unhealthy food and beverage marketing, across all settings and media, will minimise shifts of marketing practices from regulated to unregulated media, settings and times, as was seen in the earlier phases of regulating tobacco marketing [54–56].

### Tailor the Legislative Design to Media, Settings or Techniques

Given the inadequacies of a ‘child-directed’ approach, governments need to consider how each setting, media and technique will be regulated, as a blanket ban on all unhealthy food and beverage marketing is unlikely to be a realistic alternative. To ascertain the most appropriate legislative design, each media, setting and technique can be assessed to understand the practicalities of effectively restricting the exposure of unhealthy food and beverage marketing in the specific context.

Typically, most marketing practices can be categorised into a setting-based, time-based, media-based or content-based design approach (see Table 1). For example, in settings where there is frequently a high number (not proportion) of children present at all times (e.g. public spaces or retail environments), a whole-of-setting ban on unhealthy food and beverage marketing should be considered. For media types that are more linear in nature (e.g. broadcast), a time-based approach may be utilised, restricting all unhealthy food and beverage marketing between times when large numbers (not proportions) of children are in the audience. In other media, where the audiences are highly mixed between adults and children and when the advertising models are complex and difficult to control during specific hours or times (e.g. online media), a media-based restriction that covers the entire medium, at all times, should be considered. For any marketing media, settings or techniques where it would be ineffective or inappropriate to be regulated with a time-, media- or setting-based legal design, a content-based regulation could be considered. A content-based design would ban unhealthy food and beverage marketing where the creative content of the marketing message is considered to be ‘directed to children’. This should not be considered as the primary legal design but rather a default design approach that captures any unhealthy food and beverage marketing that children are exposed to that is not captured in the setting-, time- and media-based design approaches. For example, this may be appropriate for marketing of product packaging of unhealthy foods and beverages or during television times that are outside of the specified time-based restrictions. Table 1 categorises these different approaches to regulatory design.

**Table 1:** Types of legal design

**Time-based restrictions:** where the medium can be restricted by time	***Policy design****:* restrict advertising between a certain time period where a large number of children are in the audience ***Examples:*** broadcast media such as television, cinema, radio
**Setting-based restriction:** where the setting is one where children commonly frequent or has a mixed audience of children and adults	***Policy design:*** restrict all unhealthy food and beverage marketing in those specified settings (regardless of whether it is directed to children) ***Examples:*** places commonly frequented by children (schools, child services, playgrounds, children’s sports, etc.); public spaces, transport and events; outdoor marketing (billboards etc.); retail environments (point of sale marketing techniques)
**Media-based restriction:** where the medium has a highly mixed audience of children and adults and/or where advertising models are complex	***Policy design:*** restrict all unhealthy marketing disseminated through specified media (regardless of whether it is directed to children) ***Examples:*** online digital environments; direct mail, unsolicited documents or any other direct marketing to children or which has the intent to influence children’s consumption of marketed products
**Content-based restriction:** if a time-, setting- or media-based restriction is an inappropriate design, or for where exemptions are made to such restrictions, marketing that is considered to be ‘directed to children’ can be banned	***Policy design****:* restrict marketing where the creative content is ‘child-directed’ or ‘child-appealing’ ***Examples:*** product packaging (on- or in-pack promotions); hours outside of a time-based ban for television; products that are promoted with unhealthy foods or beverages (e.g. meals with toys)

### Food or Nutrient Classification Systems

Food and nutrient classification systems are those that scientifically determine whether a food may or may not be marketed under a relevant food marketing regulatory regime. Many countries have adopted a nutrient-based classification, often with pre-defined food categories that are considered within scope of the legal measures [18••]. Governments can adapt regional nutrient profile models developed by the WHO [57–59] or apply the NOVA classification system [60], which categorises foods according to the levels of processing. In reality, most countries develop their own systems and in doing so end up using a food classification model that is not comprehensive enough to cover all foods and beverages that are harmful to health. For example, almost all countries do not restrict the marketing of beverages containing non-nutritive sweeteners, despite recommendations that they be banned within WHO nutrient profile models [58, 59, 61] and the increasing evidence that these products increase the risk of adverse health outcomes [61]. Weaker food classification models can weaken the strength of the legislation and the intended effect.

The reasons for the adoption of weaker food classification models again likely relate to the powerful opposing force of the food and beverage industry. To protect against both domestic or international legal and trade challenges, governments must ensure that the evidence base for the foods chosen is scientifically robust and not unjustifiably discriminatory against certain food groups or products, particularly those from other countries [14•, 62]. Where certain food groups are exempted from being in scope of the law by a government, for example, certain types of dairy products, those exemptions must be scientifically justified and not be arbitrary or discriminatory in nature [14•, 62]. A strong classification system that aligns with national dietary guidelines is critical, but the WHO models are a strong starting point because they are evidence-based.

## Balancing a Comprehensive Legislative Approach with Practical Implementation

The WHO guidance promotes adopting a comprehensive approach to regulating unhealthy food and beverage marketing [10, 11, 13, 63]. In addition, lessons from regulating tobacco control have shown that a comprehensive law that covers all components and forms of tobacco promotion and advertising is the most effective strategy to protect population health [54–56]. However, in reality, very few countries have regulated the full scope of marketing practices and techniques across all media and settings where children are exposed to unhealthy food marketing [13, 14•, 18••]. It can be difficult for governments to introduce a full suite of restrictions with one piece of legislation because of limited capacity and resources for implementation across multiple media and settings. It can also be difficult to garner the political support to restrict marketing in a variety of different ways across media and techniques. But, introducing separate and fragmented legislation for different settings or media, such as schools, broadcast, online environments or retail environments, is likely to increase the risk of industry shifting marketing spends from regulated to unregulated media to ensure sales are not impacted [54–56]. This would reduce the overall impact of the legislation as children remain exposed to high volumes of unhealthy food and beverage marketing.

Despite this, the fact that no country around the world has introduced a law that covers all unhealthy food and beverage marketing through all media and settings demonstrates the practical challenges of doing so. One way to address this challenge is for governments to pass an overarching enabling law that creates a legal mandate for restricting unhealthy food and beverage marketing and outlines the key terms and definitions of the legislative approach (e.g. what age will be protected, what mediums/settings will be covered etc.). The government can then choose to operationalise the legislation through different regulations in a time-bound stepped approach. It is imperative to include the timeframes for implementation in the overarching legislation to signal to industry that marketing on certain media, settings and techniques will occur.

As an example, the Chilean Food Labelling and Advertising Law was approved and published in July 2012, and the law established a period of ‘1 year after its official publication’ to publish regulations outlining the operational details of the law’s implementation [24]. Stakeholders involved in the process indicated that separating out the legislative process from the regulatory design enabled the government to establish a legal milestone with a political consensus. The passage of the law demonstrated agreement that there was political will and a ‘need to regulate’, which reduced the ability of actors who opposed the law to affect the regulatory process. It also created more flexibility for the government, as the implementation details were incorporated into the regulations which could be more easily changed to keep up with developments in marketing tactics [24].

Ultimately, countries need to choose an approach that works best for their political, social and commercial contexts, keeping in mind that the final goal is to create a strong comprehensive legislative response that protects children from unhealthy food and beverages marketing across *all* media, settings and techniques.

## Monitoring and Enforcement

### Strengthening Processes for Monitoring Compliance

Monitoring is essential to identify breaches of the law (compliance) and to understand if the law is having its intended effect (evaluation). This section focuses on monitoring for compliance as it is a key barrier to effective policy implementation [64]. Most countries that restrict marketing of unhealthy foods and beverages use a public complaints system for compliance monitoring, for example, Norway and the UK. This is where the general public can file a complaint about examples of marketing practices that potentially breach the legislation and accompanying regulation to a designated complaint-handling body. The complaint-handling bodies manage the complaints process by taking the decision to a complaints board or an advisory panel who make a decision on whether the complaint will be upheld and if the advertiser responsible is penalised. While a public complaint system should be not be relied upon as the only monitoring mechanism, when it is used, it is important that it is independent, transparent, credible and free from industry influence [65].

A robust compliance system requires the government to carry out its own monitoring of compliance with ongoing auditing of marketing practices across all media and settings covered by the legislation. This is difficult in practice as monitoring is complex and resource intensive. More work is required to identify novel and low-resource mechanisms for monitoring unhealthy food and beverage marketing, and where possible, existing laws and government processes should be leveraged. Monitoring systems should be expressly outlined in the legal framework to ensure each system is resourced adequately and the appropriate authority is given to the system to act.

### Increasing Enforcement Powers with Appropriate Penalties

Penalties for non-compliance need to be consistently given and also must be sufficient in scale to adequately deter actors from non-compliance with the legislation [65]. Governments use a range of enforcement mechanisms including incentives to encourage compliance but also disincentives that are stricter such as fines to increase the rate of compliance with the legislative system [65]. For example, in South Korea, advertisers who breach television regulations for food marketing are liable for fines up to ten million won (almost USD $10,000) although this has been criticised as being too low [66], and in Chile, breaches of the Food Labelling and Advertising Law are liable for penalties including reprimands, fines or prohibition from selling an advertised product.

Generally, penalties for non-compliance of food marketing laws need to be stronger and more rapidly issued. Often, a marketing message has already had its intended effect by the time the process of complaint, judgement and enforcement has been completed (particularly in the digital environment). Penalties may take a variety of forms including requiring an offending company to modify and withdraw the offending marketing campaign across all media and settings in scope or disallowing an offending company to use a particular marketing channel for a specified time or sell or market a certain product. Naming and shaming the offending company or requiring an offending company to pass a pre-clearance process before advertising any further campaigns can also have a strong impact. If the legislative mandate allows, imposing criminal or civil charges and accompanying liability on company officers or directors would also be an effective deterrent.

Governments should consider what person or entities on the entire marketing chain (initiators, producers and publishers, people engaged with marketing (e.g. influencers) or people who receive or facilitate marketing (sporting clubs receiving sponsorships)) will be legally obliged to comply with the legislation. The Framework Convention on Tobacco Control requires that national laws put in place to restrict tobacco advertising must ensure all persons and entities involved in the marketing chain are responsible for upholding the advertising laws [55, 56]. Governments may impose a duty on online platforms, who collect and sell children’s data, so that they cannot collect such data for commercial gain and request that prohibited content is removed or reasonable efforts are taken to disable access to it, where technically possibly. The penalties can be scaled depending on the level of responsibility the actor has for the breach.

## Conclusion

A robust legislative system that effectively restricts exposure and power of unhealthy food and beverage marketing to children would address the following key components: define children as up to 18 years of age; accurately capture the full extent of marketing that children are exposed to, regardless of the intended audience, in the scope of the law and accompanying regulation including the full range of settings, media and techniques they are exposed to; use a time-, media-, setting- and content-based design depending on the type of marketing that is being regulated instead of a purely ‘child-directed’ approach; and use an evidence-based system to define which food and beverages (including master brands) are ‘permitted’ and ‘not permitted’ under the regulatory framework.

The strategy behind implementing a comprehensive approach at one time or in stages will depend on the resources and political will in each country, but any stepped implementation should be time-bound and enabled by the overarching legislation to ensure momentum is not lost. Efficient monitoring for compliance and adequate deterrents for non-compliance with penalties that are relevant to the food and beverage industry are just as important as the design of the law to ensure the law reaches its stated objectives.

## Data Availability

The data that support the findings of this study are available from the corresponding author, [FS], upon reasonable request.
